# Does my patient have sex ? - Mental health professionals’ understanding of sexual health issues among their patients

**DOI:** 10.1192/bjo.2021.685

**Published:** 2021-06-18

**Authors:** Nalin Hettiarachchi, Praveen Kumar, vikramraj balasundaram

**Affiliations:** 1 Russells Hall Hospital; 2 Bushey Fields Hospital

## Abstract

**Aims:**

To assess the level of understanding and difficulties encountered when obtaining sexual health details of their patients among mental health clinicians.

**Background:**

People with mental health problems, especially those treated with psychiatric medication experience greater rates of sexual difficulties than those in the general population. Mental health practitioners need to examine personal beliefs and attitudes about sexuality among people with mental health problems. Providing information about sexuality and sexual practice benefits and enhances the quality of life of people with mental health problems. Therefore taking a sexual history should be an integral part of psychiatric assessment.

**Method:**

An online survey consisted of 17 questions to cover 3 areas of objectives mentioned above was created using Survey Monkey. A link to the survey was emailed to all the clinicians who perform psychiatric assessments. Response collection and data analysis was performed by the trust IT team.

**Result:**

Total of 54 clinicians participated in the survey representing nurses, junior, middle grade doctors and consultants. Almost all stated that mental health patients have capacity to make appropriate decisions about their sexual behaviour patterns. 43% thought people with mental health problems don't have similar patterns of sexual behaviour compared to people without mental health problems. 11% stated that people with mental health problems do not experience greater rates of sexual difficulties than those in the general population. Nearly a third did not believe that telling patients about potential sexual side effects may lead to poor compliance. Nearly 70% stated taking a sexual history should be an integral part of psychiatric assessment. 44% reported lack of knowledge and skills when talking about sexual health and 33% avoided asking about sexual health due to lack of knowledge. Half of the clinicians avoided asking about sexual health due to the fear of embarrassing or causing distress to patients while 16% avoided asking about sexual health due to self-embarrassment. 65% talk about sexual health issues only if patients brought them up.

During last 3 clinical encounters majority never asked about sexual difficulties, high risk behaviour and drug side-effects related to sexual difficulties. A significant proportion of clinicians never asked about contraception from their female clients.

**Conclusion:**

Survey revealed majority of mental health clinicians lack understanding and skills about sexual health issues highlighting the importance of raising awareness among clinicians about sexual health issues.

